# Key Subdomains of Cerebral Dopamine Neurotrophic Factor Regulate Its Protective Function in 6-Hydroxydopamine-Lesioned PC12 Cells

**DOI:** 10.1089/dna.2023.0215

**Published:** 2023-11-10

**Authors:** Hao Liu, Haibin Dong, Chunxiao Wang, Wenjuan Jia, Guangqiang Wang, Hua Wang, Lin Zhong, Lei Gong

**Affiliations:** The Affiliated Yantai Yuhuangding Hospital of Qingdao University, Yantai, China.

**Keywords:** CDNF, Parkinson's disease, neurotrophic factor, secretion, endoplasmic reticulum stress

## Abstract

Cerebral dopamine neurotrophic factor (CDNF) is a unique neurotrophic factor (NTF) that has shown significant neuroprotective and neurorestorative functions on midbrain dopaminergic neurons. The secondary structure of human CDNF protein contains eight α-helices. We previously found that two key helices, α1 and α7, regulated the intracellular trafficking and secretion of CDNF protein in different manners. The α1 mutation (M1) induced most CDNF proteins to reside in the endoplasmic reticulum and little be secreted extracellularly, while the α7 mutation (M7) caused the majority of CDNF proteins to be secreted out of the cells and little reside in the cells. However, the regulation of the two mutants on the function of CDNF remains unclear. In this study, we investigated the effects of M1 and M7 on the protective activity of CDNF in PC12 cells, which were treated with 6-hydroxydopamine (6-OHDA) to mimic Parkinson's disease. We found that both M1 and M7 could promote survival and inhibit apoptosis more effectively than Wt in 6-OHDA-lesioned PC12 cells. Therefore, these findings will advance our understanding of the important regulation of subdomains on the function of NTFs.

## Introduction

Parkinson's disease (PD) is a neurodegenerative disease characterized by a progressive loss of dopamine neurons in the midbrain (Samii et al., 2004). The pathogenic mechanism of dopamine neurodegeneration is complex and involves multiple processes, such as mitochondrial dysfunction, oxidative stress, neuroinflammation, autophagy, and endoplasmic reticulum (ER) stress (Roussel et al., 2013). Neurotrophic factors (NTFs), such as nerve growth factor, brain-derived NTF, and glial cell line-derived NTF, have shown specific roles in supporting the survival, differentiation, and development of neurons. Cerebral dopamine neurotrophic factor (CDNF), a novel NTF, together with its homolog mesencephalic astrocyte-derived neurotrophic factor (MANF), has shown significant neuroprotective and neurorestorative functions on midbrain dopaminergic neurons (Lindholm and Saarma, 2010; Lindholm et al., 2007).

Many studies have shown that CDNF has significant nerve repair and protection in several models of PD (Albert et al., 2021; Bäck et al., 2013; Huotarinen et al., 2018; Mätlik et al., 2017; Mei and Niu, 2014). In PC12 cells, CDNF protected cells from apoptosis caused by 6-hydroxydopamine (6-OHDA) *in vitro* (Mei and Niu, 2014). In a rat experimental model of PD, injection of CDNF into the substantia nigra significantly restored the function and prevented the degeneration of dopaminergic neurons (Mätlik et al., 2017). In addition, CDNF is currently in Phase I/II clinical trials for PD (Huttunen and Saarma, 2019). Therefore, CDNF has great application prospects in the prevention and treatment of PD.

Unlike other classical NTFs, CDNF and MANF belong to a newly characterized family with a distinct structure and function (Jӓntti and Harvey, 2020; Lindström et al., 2013). The human CDNF protein contains 187 amino acids and the mature protein is 18 kDa in molecular weight. The secondary structure of CDNF contains eight α-helices. The three-dimensional structure shows that they have two independently folded domains, the N-terminal saposin-like domain and the C-terminal SAP (SAFA/B, Acinus and PIAS) domain, connected by a flexible linker (Jӓntti and Harvey, 2020; Lindström et al., 2013).

The N-terminal domain contains five α-helices (α1–α5) and is structurally homologous to saposins, which are cysteine-rich proteins that are known to interact with lipids and membranes (Hoseki et al., 2010). Thus, it is plausible that the N-terminal domain may mediate the interaction of CDNF with membranes and initiate its intracellular signaling pathway. Meanwhile, the C-terminal domain contains three α-helices (α6–α8) and is structurally similar to the SAP domain of the Ku70 protein. Through this SAP domain, Ku70 can interact with Bax, a proapoptotic protein, to inhibit cell apoptosis (Hellman et al., 2011; Lee et al., 2012). In addition, the C-terminus of human CDNF contains an ER retention sequence KTEL (Lys-Thr-Glu-Leu), which closely resembles the classical ER retention sequence KDEL (Lys-Asp-Glu-Leu) that can interact with KDEL receptors in the ER (Raykhel et al., 2007).

Many studies have shown that CDNF can act as an ER-resident protein to modulate the unfolded protein response (UPR) during ER stress (Henderson et al., 2013; Jӓntti and Harvey, 2020; Norisada et al., 2016). Therefore, unlike the other classical NTFs that are usually secreted extracellularly and exert neurotrophic activity by binding to membrane receptors, CDNF may not only be secreted extracellularly to bind with still-unidentified plasma membrane receptors but also can reside in the ER to maintain ER homeostasis and protect cells against ER stress (Albert and Airavaara, 2019; Lindahl et al., 2017; Lindholm and Saarma, 2021; Liu et al., 2015a). This action mode may be important for the neurotrophic activity of CDNF on midbrain dopaminergic neurons.

For CDNF, we have previously found two key helices, α1 and α7, which could regulate the intracellular trafficking and secretion of CDNF protein in different manners. In previous studies, we constructed eight α-helix mutations of CDNF by inserting proline into the respective position to destroy the corresponding α-helix structure.

The results showed that the α1 mutation (M1) induced most CDNF proteins to reside in the ER and fail to be secreted extracellularly, while the α7 mutation (M7) caused the majority of CDNF proteins to secrete out of the cells (Liu et al., 2019; Liu et al., 2015b, Sun et al., 2011). Thus, α1 and α7 are important in regulating its trafficking and secretion of CDNF. However, whether they further affect the neuroprotective activity of CDNF remains unclear. This study aimed to investigate the effects of α1 and α7 subdomains on the neuroprotective function of CDNF in PC12 cells after 6-OHDA administration.

## Materials and Methods

The study protocol was approved by Ethics Committee of the Yantai Yuhuangding Hospital.

### Reagents

Dulbecco's modified Eagle medium (DMEM; 11960-044), fetal bovine serum (12484-010), horse serum (26050070), penicillin–streptomycin (15140-122), and Lipofectamine 3000 (L3000008) were all obtained from Invitrogen. Hemagglutinin (HA)-Tag (6E2) Mouse mAb (2367), p44/42 ERK1/2 (9102), and phospho-p44/42 ERK1/2 (Thr202/Tyr204) (9101), Alexa Fluor 488-conjugated goat anti-mouse IgG antibody (4408), and Alexa Fluor 594-conjugated goat anti-rabbit IgG antibody (8889) were obtained from Cell Signaling Technology. Rabbit antibody against the ER marker calnexin (SAB4503258) and rabbit antibody against the Golgi apparatus marker TGN38 (T9826) were from Sigma.

GAPDH (ab8245), tyrosine hydroxylase (TH) (ab137869), and NF-κB p65 antibodies (ab16502) were obtained from Abcam. Phospho-NF-κB p65 (Ser536) (AF2006), phospho-pan-Akt1/2/3 (Ser473) (AF0016), and pan-Akt1/2/3 antibodies (AF6261) were obtained from Affinity Biosciences. Tubulin (10068-1-AP), CHOP (15204-1-AP), and GRP78 (11587-1-AP) antibodies were obtained from Proteintech. Radioimmunoprecipitation assay (RIPA) lysis buffer (P0013B) and bicinchoninic acid (BCA) assay kit (P0010) were obtained from Beyotime Biotechnology. Cell Counting Kit-8 (CCK-8; E606335) was obtained from BBI. Annexin V-FITC/PI Apoptosis Detection Kit (KGA108) was obtained from Keygen Biotech. All other compounds were from Sangon Biotech.

### Cell culture and 6-OHDA treatment

PC12 (clone 615) cells were maintained in DMEM with 5% fetal bovine serum and 10% horse serum, supplemented with 100 U/mL penicillin–streptomycin and 2 mM glutamine plus 200 mg/mL G418 at 37°C with 5% CO_2_. Upon reaching 80% confluence, the cells were treated with varying doses (0, 20, 40, 60, and 80 μM) of 6-OHDA for 24 h. Then, cell viability was measured using the CCK-8 assay to determine the optimal concentration of 6-OHDA to mimic PD in PC12 cells.

### Cell viability assay

PC12 cells were seeded in 96-well plates at a density of 1 × 10^3^ cells per well. The cells were first washed with DMEM, and 10 μL CCK-8 solution were added into each well. After incubation for 4 h, the absorbance value was measured at 450 nm using a spectrophotometer (ThermoFisher, NY).

### Cell transfection

The plasmids of Wt (wild-type CDNF), M1 (proline inserted between L44 and N45 to destruct α1), and M7 (proline inserted between L151 and H152 to destruct α7), which have been constructed as full-length cDNA, were subcloned into the pcDNA3.1neo expression vector, and the HA epitope tag was added to the 3′ end of the CDNF through PCR (Liu et al., 2019). Proline is an α-helix breaker in terms of its hydrogen bonding capability and the bulkiness of its side chain near the backbone (Barlow and Thornton, 1988). In cell transfection, PC12 cells were divided into five groups: control group (Con), wherein the cells were cultured without treatment; 6-OHDA group, wherein the cells were transfect with null pcDNA3.1neo vector and treated with 6-OHDA for 24 h; and Wt, M1, and M7 groups, wherein the cells were transfected by Wt, M1, and M7, respectively, and treated with 6-OHDA.

Cell transfection was performed using Lipofectamine 3000 according to the manufacturer's instructions. Briefly, 5 μg DNA and 10 μL P3000 reagent were diluted in 125 μL OptiMEM. And 10 μL Lipofectamine 3000 reagent were diluted in another 125 μL OptiMEM. Then, the diluted DNA was added to the diluted Lipofectamine 3000 Reagent and mixed gently. After incubation for 15 min, the mixture was added to the cell cultures. After incubation for 6 h, the culture medium was changed to complete medium and 6-OHDA was added in the medium at same time. After incubation for 24 h, the cells were then harvested for subsequent experiments.

### Western blot

Protein was extracted from the cells using RIPA lysis buffer with 1 mM phenylmethanesulfonyl fluoride and 2% protease inhibitor cocktail. The protein concentration was determined using a BCA assay kit. After separation by sodium dodecyl sulfate-polyacrylamide gel electrophoresis, the proteins were transferred onto polyvinylidene difluoride membranes. The membranes were then blocked in 10% milk in Tris-buffered saline with Tween 20 and incubated with the primary antibodies, including anti-HA (1:3000), anti-TH (1:1000), anti-CHOP (1:800), anti-GRP78 (1:800), anti-p44/42 ERK1/2 (1:1000), anti-phospho-p44/42 ERK1/2 (1:1000), anti-NF-κB p65 (1:1000), anti-phospho-NF-κB p65 (1:1000), anti-pan-Akt1/2/3 (1:1000), anti-phospho-pan-Akt1/2/3 (1:1000), anti-Tubulin (1:5000), and anti-GAPDH (1:5000), and subsequently incubated with the secondary antibody. The protein bands were visualized using an enhanced chemiluminescence substrate. The intensity of immunoreactive bands was scanned and quantified with Image-Pro Plus (Media Cybernetics, Inc., Bethesda, MD).

### Immunocytofluorescence and quantitative analysis

The immunocytofluorescence staining was performed as described before (Liu et al., 2019). Briefly, PC12 cells were subcultured on poly-d-lysine-coated glass coverslips and transfected with Wt, M1, and M7, respectively, and treated with 6-OHDA. After 24 h, cells were fixed and permeabilized, then blocked by 1% BSA, and incubated with HA antibody and calnexin antibody or TGN38 antibody followed by Alexa Fluor 488-conjugated goat anti-mouse IgG antibody and Alexa Fluor 594-conjugated goat anti-rabbit IgG antibody. Fluorescence images were acquired by Zeiss Axio Observer7 microscope (Carl Zeiss, Oberkochen, Germany). The proportion of colocalization was quantitated using ZEN software. Approximately 25 cells were examined at random and three independent experiments were repeated.

### Flow cytometry

PC12 cells were digested with trypsin and washed with phosphate-buffered saline twice, and 1 to 5 × 10^5^ cells were collected. Next, 500 μL of binding buffer were added to suspend the cells, and 5 μL of Annexin V-FITC were added and mixed well. Then, the cells were incubated with 5 μL propidium iodide (PI) dye solution for 5 to 15 min at room temperature in the dark. Samples were detected using a flow cytometer (CytoFLEX; Beckman, CA) within 1 h.

### Statistical analyses

Statistical analyses were performed using SPSS 24.0 software (IBM, Armonk, NY). Shapiro–Wilk analyses were used to test the data distribution normality and all experimental data have normal distribution, which are expressed as the mean ± standard deviation. Comparisons between results of multiple groups were performed using analysis of variance analysis. Statistical significance was set at *p* < 0.05. All experiments in this study were repeated thrice and *n* = 3 for each group. The results of three repetitions of each experiment are multiple measurements made by the same operator, in the same laboratory, using the same instrument, and at a specific time.

## Results

### The secretion and sublocalization of CDNF protein in 6-OHDA-lesioned PC12 cells

**FIG. 1.**
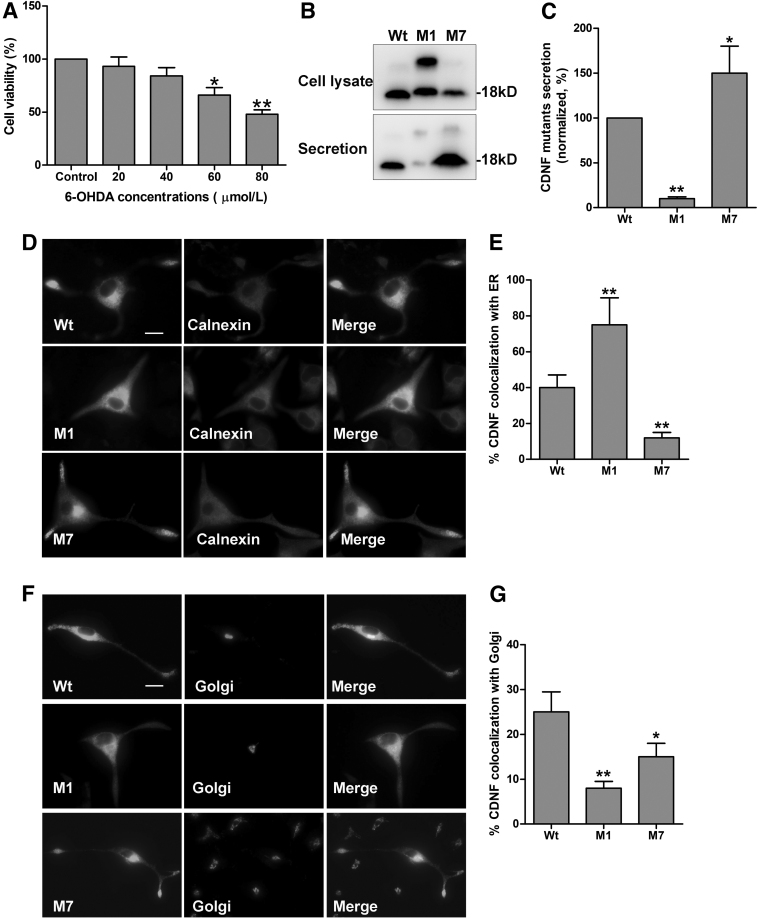
Effect of 6-OHDA on the secretion and sublocalization of CDNF protein in PC12 cells. **(A)** CCK-8 assay showed the cell viability of PC12 cells treated with varing concentration (0, 20, 40, 60, and 80 μM) of 6-OHDA for 24 h. The value of the 0 μM group cells was set to 100%, to which the values of the other groups were normalized to. Data were presented as the mean ± SD determined from analysis of three independent experiments. Comparisons between the groups were performed using ANOVA and Dunnett's *t*-test was used as *post hoc* test (*p < 0.05 or **p < 0.01 vs. 0 μM group). **(B)** PC12 cells were transfected with Wt, M1, or M7. Lysate and medium were prepared and analyzed by immunoblotting with anti-HA antibody. **(C)** The value of medium versus cell lysate from Wt was set to 100%. The value of secretion of M1 and M7 was normalized to Wt. **(D)** Colocalization of the ER marker, calnexin, was visualized by costaining with anti-HA and anti-calnexin antibodies. Scale bar, 10 μm. **(F)** Colocalization of CDNF and the Golgi apparatus marker, TGN38, was visualized by co-staining with anti-HA and anti-TGN38 antibodies. Scale bar, 10 μm. **(E, G)** Quantitative analysis of the proportion of colocalization between CDNF and ER or Golgi. Data are presented as mean ± SD determined from analysis of three independent experiments. Comparisons between the groups were performed using ANOVA and Dunnett's *t*-test was used as *post hoc* test (*p < 0.05 or **p < 0.01 vs. Wt group). 6-OHDA, 6-hydroxydopamine; CDNF, cerebral dopamine neurotrophic factor; ER, endoplasmic reticulum; HA, hemagglutinin; M1, α1 mutation; SD, standard deviation.

To mimic PD in PC12 cells, we first determined the optimal concentration of 6-OHDA added in the medium of PC12 cells. The cells were treated with varying doses (0, 20, 40, 60, and 80 μM) of 6-OHDA for 24 h. The results showed that the cell viability was reduced (100%, 93%, 84%, 66%, and 48%, respectively) with increasing concentrations of 6-OHDA (Fig. 1A). And the cell viability of 60 and 80 μM showed significant differences comparing with that of 0 μM (*p < 0.05, **p < 0.01). According to the cell status, 60 μM 6-OHDA treatment for 24 h was used in subsequent experiments.

In this study, we detected the expression and secretion of CDNF in 6-OHDA-lesioned PC12 cells. Wt, M1, and M7 were transfected into PC12 cells, respectively, and treated with 60 μM 6-OHDA for 24. Then, both secretion and cell lysate were collected for immunoblotting. The secretion level is represented by the ratio of secreted proteins to cell lysate proteins. As shown in Figure 1B and C, all Wt, M1, and M7 proteins could be detected in the cell lysate. The secretion level of M1 protein decresed significantly, while the secretion level of M7 protein increased obviously compared with Wt (*p < 0.05, **p < 0.01). Therefore, the CDNF protein and two mutants secrete in different manners in 6-OHDA-lesioned PC12 cells.

Furthermore, we assessed sublocalization in the ER and Golgi apparatus of CDNF protein by immunocytofluorescence. We found that M1 protein mostly colocalized with calnexin (a marker of ER), but little with TGN38 (a marker of trans-Golgi network) compared with Wt (Fig. 1D, E). However, M7 protein showed less localization in both ER and Golgi apparatus compared with Wt (Fig. 1F, G). Then, the sublocalization of CDNF and two mutants are different. Most M1 protein reside in the ER, but most M7 protein secrete out of the cells in 6-OHDA-lesioned PC12 cells.

### Regulation of α1 and α7 on cell survival and key protein expression involved in PD

**FIG. 2.**
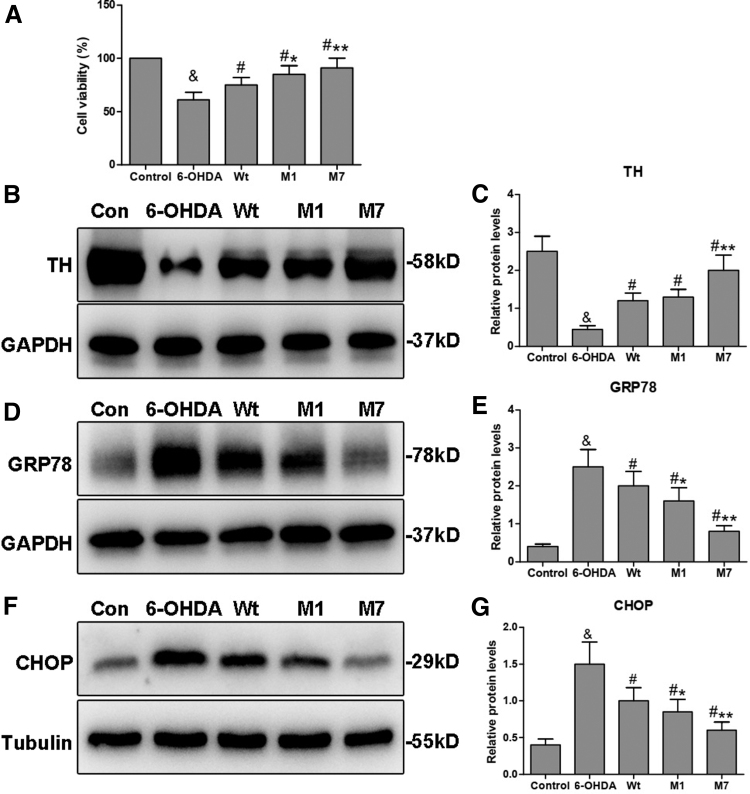
Regulation of α1 and α7 on cell survival and key protein expression involved in PD. **(A)** Cell viability was measured using the CCK-8 method. The value of the control group, which was without treatment, was set as 100%, to which the values of other groups were normalized. **(B, C)** Western blot analyses and quantification of the levles of TH protein in PC12 cells. **(D, E)** Western blot analyses and quantification of the levles of GRP78 protein in PC12 cells. **(F, G)** Western blot analyses and quantification of the levels of CHOP protein in PC12 cells. Data were presented as the mean ± SD determined from analysis of three independent experiments. Comparisons between the groups were performed using ANOVA and Bonferroni test was used as *post hoc* test (^&^*p* < 0.05 vs. Control group; ^#^*p* < 0.05 vs. 6-OHDA group; *p < 0.05 or **p < 0.01 vs. Wt group). CCK-8, Cell Counting Kit-8; PD, Parkinson's disease; TH, tyrosine hydroxylase.

CCK-8 assay was used to determine the effect of CDNF mutants on cell survival. As shown in Figure 2A, the cell viability was significantly reduced in the 6-OHDA group (61%) compared with that in the control group (100%; ^&^*p* < 0.05), but the cell viability was significantly increased when individually transfected with Wt, M1, or M7 compared with that in the control group (^#^*p* < 0.05). The M1 group (83%) and M7 group (91%) both showed elevated levels of cell viability compared with the Wt group (75%) (*p < 0.05, **p < 0.01). Thus, these results indicate that the M1 and M7 can preserve cell survival destroyed by 6-OHDA and their preservation may be more effective than Wt.

TH is a key neuron-specific enzyme required for dopamine synthesis. We detected the levels of TH protein by Western blot analysis. The results showed that 6-OHDA administration significantly reduced the level of TH protein when compared with the control group (^&^*p* < 0.05). CDNF overexpression (Wt, M1, and M7) could significantly upregulate the TH level compared with the 6-OHDA group (^#^*p* < 0.05). Notably, the level of TH protein in the M1 group was similar to that in the Wt group. However, in the M7 group, the TH level was significantly higher than that in the Wt group (**p < 0.01; Fig. 2B, C). Thus, M1 does not affect the role of CDNF on reversing the level of TH protein in 6-OHDA-lesioned PC12 cells, but this effect may be more effective for M7.

ER stress is an important pathological process in PD. It has been reported that CDNF can act both as a secreted NTF and as an ER stress response protein (Liu et al., 2015a). Therefore, we evaluated the expression of GRP78 and CHOP, which are chaperones involved in ER stress. As shown in Figure 2D–G, GRP78 and CHOP were upregulated in the 6-OHDA group compared with the control (^&^*p* < 0.05), whereas CDNF transfection significantly reduced their levels compared with the 6-OHDA group (^#^*p* < 0.05). When compared with the Wt group, both M1 and M7 groups showed significantly decreased levels of GRP78 and CHOP (*p < 0.05, **p < 0.01). Thus, both M1 and M7 may relieve ER stress more effectively in 6-OHDA-lesioned PC12 cells.

### Regulation of α1 and α7 on cell apoptosis in 6-OHDA-lesioned PC12 cells

**FIG. 3.**
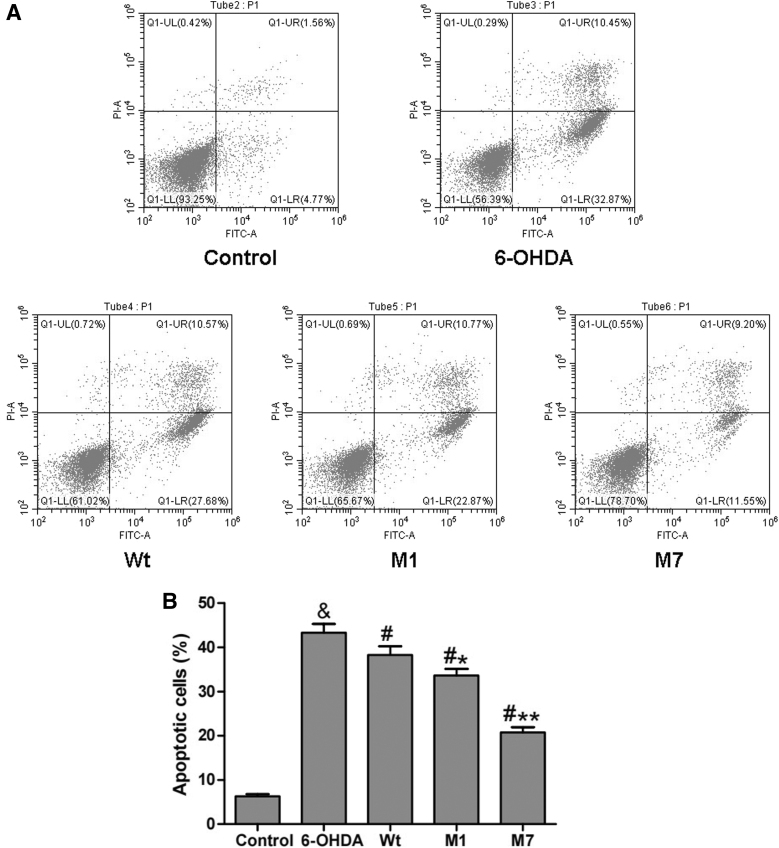
Regulation of α1 and α7 on cell apoptosis in 6-OHDA-lesioned PC12 cells. **(A)** Representative images of flow cytometry. **(B)** The percentage of apoptotic cells was quantified. Data were presented as the mean ± SD determined from analysis of three independent experiments. Comparisons between the groups were performed using ANOVA and Bonferroni test was used as *post hoc* test (^&^*p* < 0.05 vs. Control group; *^#^p* < 0.05 vs. 6-OHDA group; *p < 0.05 or **p < 0.01 vs. Wt group).

Furthermore, we examined the effect of CDNF mutants on cell apoptosis through flow cytometry. The results of Figure 3 revealed that 6-OHDA treatment greatly upregulated cell apoptosis (43.32%) compared with the control group (6.33%; ^&^*p* < 0.05). In all the Wt, M1, and M7 groups, the cell apoptosis was reduced compared with the 6-OHDA group (^#^*p* < 0.05). Moreover the percentage of apoptotic cells of the M1 group was 33.64%, which was less compared with the Wt group (38.25%; *p < 0.05). And the percentage of apoptotic cells of the M7 group was 20.75%, which has a significantly decreased level compared with the Wt group (**p < 0.01). These results indicate that both M1 and M7 can protect cells against apoptosis more effectively than Wt CDNF in 6-OHDA-lesioned PC12 cells.

### Regulation of α1 and α7 on the signal pathways involved in PD

**FIG. 4.**
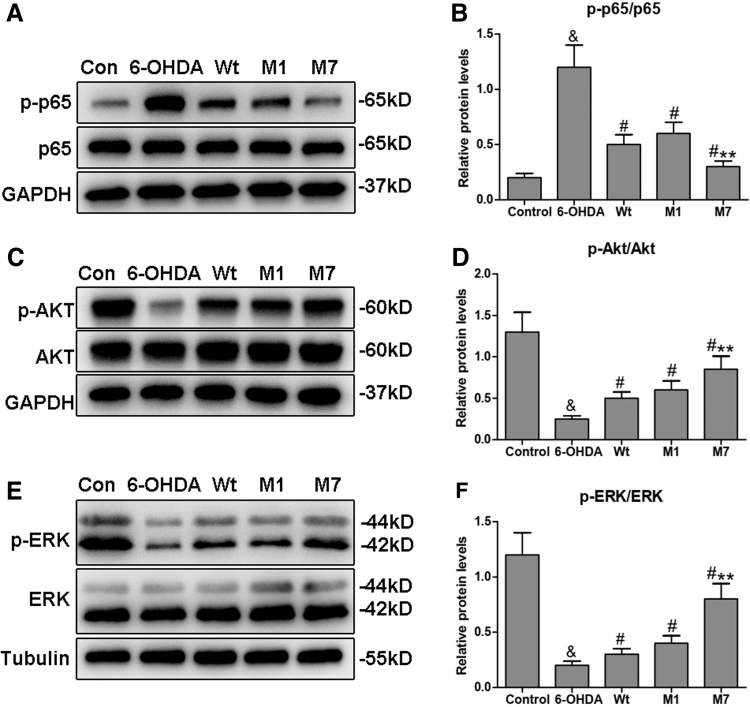
Regulation of α1 and α7 on the signal pathways involved in PD. **(A, B)** Western blot analyses and quantification of the levels of p65 and p-p65. **(C, D)** Western blot analyses and quantification of the levels of Akt and p-Akt. **(E, F)** Western blot analyses and quantification of levels of ERK and p-ERK. Data were presented as the mean ± SD determined from analysis of three independent experiments. Comparisons between the groups were performed using ANOVA and Bonferroni test was used as *post hoc* test (^&^*p* < 0.05 vs. Control group; *^#^p* < 0.05 vs. 6-OHDA group; **p < 0.01 vs. Wt group).

To explore the effect of α1 and α7 on the signal pathways involved in PD, we first investigated the activity of the NF-κB pathway. As shown in Figure 4A and B, the 6-OHDA group had elevated p65 phosphorylation levels compared with the control group (^&^*p* < 0.05). However, CDNF transfection markedly decreased the phosphorylation levels of p65 compared with the 6-OHDA group (^#^*p* < 0.05). In addition, the phosphorylation levels of p65 in the M1 group was similar as the Wt group, while the M7 group showed lower phosphorylation levels of p65 compared with the Wt group (**p < 0.01).

Next, we studied the changes in the PI3K-Akt and ras-mitogen-activated protein kinase (MAPK) pathways, which can promote neuronal survival and stimulate neurite outgrowth in PD (Brunet et al., 2001). As shown in Figure 4C–F, the phosphorylation levels of Akt and ERK decreased in the 6-OHDA group compared with the control group (^&^*p* < 0.05). In contrast, CDNF transfection significantly activated Akt and ERK phosphorylation in the Wt, M1, and M7 groups compared with the 6-OHDA group (^#^*p* < 0.05). Akt and ERK phosphorylation of the M1 group did not show differences with the Wt group. However, in the M7 groups, Akt and ERK phosphorylation were markedly increased compared with that of the Wt group (**p < 0.01). These findings strongly suggest that the α7 subdomain can efficiently regulate the NF-κB, Akt, and ERK pathways to enhance the protective effects of CDNF.

## Discussion

CDNF has been recognized as an important potential therapeutic NTF for PD (Tang et al., 2017). As a secreted growth factor, CDNF protein is secreted by the classic ER-Golgi pathway (Sun et al., 2011). In our previous studies, we found that two key subdomains, α1 and α7, could regulate the intracellular trafficking and secretion of CDNF with different manner, in which M1 induced most CDNF proteins to reside in the ER and fail to be secreted extracellularly, while the α7 mutation caused the majority of CDNF proteins to secrete out of the cells. However, whether these subdomains affect the protective function of CDNF in PD remains unknown. First, we explored the expression and sublocalization of two mutants in PC12 cells after 6-OHDA administration.

We found that Wt, M1, and M7 could express in 6-OHDA-lesioned PC12 cells. Compared with Wt, the secretion of M1 protein reduced significantly but the secretion of M7 protein increased which were similar as them in PC12 cells that without 6-OHDA treatment in our previous studies. Furthermore, the results of sublocalization in 6-OHDA-lesioned PC12 cells showed most M1 protein reside in the ER but most M7 protein secrete out of the cells, which were also similar as that in PC12 cells that without 6-OHDA treatment. Then, 6-OHDA treatment cannot affect the secretion and sublocalization of wild-type CDNF protein and two mutants.

Second, we found both M1 and M7 could promote cell survival and inhibit cell apoptosis in PC12 cells after 6-OHDA administration and their effects may be more pronounced than Wt. The results indicate that CDNF can exert a protective effect in PC12 cells whether it resides in the cells or is secreted out of the cells. For TH protein, the results showed that M7, but not M1 could reverse its levels more effectively than Wt, while for GRP78 and CHOP, both M1 and M7 could inhibit their expression significantly than Wt. CDNF has been recognized as an ER stress response protein and ER stress plays important roles in the development of PD.

At early stages of PD, the pathways triggered by ER stress are protective. However, when the damage is extensive, ER stress will initiate apoptosis (Kovaleva and Saarma, 2021). During ER stress, GRP78 separates from the ER membrane sensors, including RNA-dependent protein kinase-like ER kinase (PERK), inositol-requiring enzyme 1 (IRE1), and activator of transcription-6 (ATF6), and initiates the UPR to restore the structure of the accumulated unfolded protein (Hendershot, 2004). However, if ER stress is prolonged and/or severe, the apoptotic signals will be initiated.

Furthermore, CHOP has been implicated as a mediator of apoptosis in the contexts of ER stress (Nishitoh, 2012). Our results indicate that CDNF may regulate the expression of GRP78 or CHOP to relieve ER stress and protect cells from apoptosis induced by 6-OHDA. Because M1 is retained in the ER, while M7 is secreted extracellularly, we originally thought that M1 would regulate the UPR pathway more strongly than Wt or M7. However, our results show that, although M7 is secreted out of the cells, it can also inhibit the expression of GRP78 and CHOP proteins and that the effect may be more pronouned than those of Wt. Then, the possible proteins interacting with CDNF subdomains need to be identified to explain the different regulation of α1 and α7 on CDNF function.

In addition, we explored the possible signal pathways involving ER stress and cell apoptosis. The NF-κB pathway is closely connected with all three branches of the UPR. Many studies have shown that the NF-κB pathway is activated in the early phase of ER stress, whereas in the later phase, subsequent UPR inhibited NF-κB signaling (Frakes and Dillin, 2017). Chen et al. (2015) found that MANF interacted with the NF-κB subunit p65 through the SAP domain to attenuate NF-κB activation induced by tumor necrosis factor alpha (TNF-α). However, the role of the SAP domain of CDNF is unknown. In this study, we found that the NF-κB pathway was activated by 6-OHDA, but was downregulated by CDNF overexpression and that the effect of M7 was more pronounced compared with Wt. Therefore, the regulation of ER stress by CDNF is independent of its localization in the ER.

The PI3K/Akt and MAPK pathways are classical signaling pathways that regulate cell proliferation, differentiation, apoptosis, and aging. In the nervous system, the activation of these pathways can inhibit neuronal apoptosis by regulating apoptosis-related proteins (Ijomone et al., 2021; Lai et al., 2016). Our results about the regulation of PI3K/Akt and ERK pathways also indicate that M7 activates the two pathways more distinctively than Wt. Therefore, the specific mechanisms for α1 and α7 in regulating its protective function of CDNF in PC12 cells still need to be further studied.

## Conclusions

Our findings indicate that α1 and α7 can upregulate the neuroprotective activity of CDNF by promoting cell survival and inhibiting cell apoptosis in PC12 cells after 6-OHDA administration. The future studies aim to identify proteins or receptors interacting with CDNF subdomains, involving regulating the activity of CDNF in PD.
